# Torsion of the Omentum: A Rare Cause of Acute Abdomen in a 14-Year-Old Boy

**DOI:** 10.1155/2018/7257460

**Published:** 2018-01-31

**Authors:** Chijioke Chinaka, Shahbaz Mansoor, Mohamed Salaheidin

**Affiliations:** Department of Surgery, Midland Regional Hospital, Longford Road, Robinstown (Levinge), Mullingar, Co. Westmeath, Ireland

## Abstract

Acute abdominal pain is a common surgical presentation, and most often, the first line of consideration is to rule out acute appendicitis; this is more so when the patient is an adolescent or within younger age group. In most cases, other differentials are considered before omental torsion. Omental torsion is a cause of acute abdominal pain and sometimes mimics acute appendicitis in its presentation. We present a case of a 14-yr-old boy who presented with acute abdomen with symptoms mimicking acute appendicitis. Laparoscopy revealed torsion of the omentum. Omentectomy and appendicectomy were done, and the child discharged four days after following a remarkable recovery.

## 1. Introduction

Omental torsion can simply be defined as twisting of the omentum on itself especially on its long axis. Frequently, this is in a clockwise direction. In this situation, venous return is compromised; thus, the distal omentum becomes congested and oedematous. As the torsion progresses, arterial occlusion leads to acute hemorrhagic infarction, and eventual necrosis of the omentum occurs [1]. Torsion of the omentum presents with acute abdominal pain which is more localised in the right iliac fossa, thus mimicking acute appendicitis or acute cholecystitis, and in the female patient, it can mimic ovarian torsion. This nonspecific presentation makes preoperative diagnosis difficult. Most cases are diagnosed intraoperatively; however, the use of computerised tomography scan and, in some cases, ultrasound scan have made preoperative diagnosis possible. Most diagnosed cases are managed surgically through omentectomy.

## 2. Case Report

A 14-year-old boy was admitted to the Emergency Department with a three-day history of severe right lower abdominal pain, anorexia, and fever. His past medical history is unremarkable apart from undescended testes that descended spontaneously at the age of 4. On physical examination, his pulse rate was 96/min, blood pressure was 132/62 mmHg, respiratory rate was 18/min, peripheral oxygen saturation was 99%, and his temperature was 37.4°C. Abdominal examination revealed guarding and tenderness on the right iliac fossa region. Lung and heart examination did not reveal any abnormality. He was given analgesia, and the blood was sent for analysis.

The laboratory report showed a normal blood analysis except the C-reactive protein which was 41. His urinalysis was normal. Abdominal ultrasonography showed fat stranding in the right iliac fossa. Appendix itself could not be visualised.

**Figure 1:**
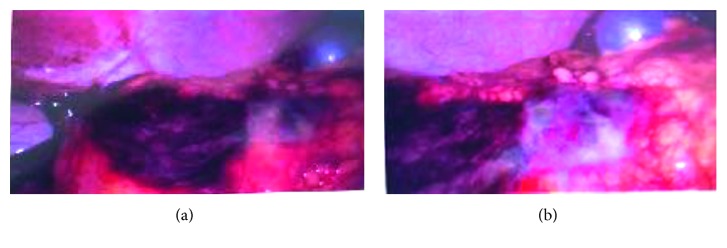
(a, b) Patchy areas of necrosis and pseudomembrane on the omentum. A look at the upper left side of (a) showed the area of attachment of the omentum with serosanguinous fluid seen at the lower end.

**Figure 2:**
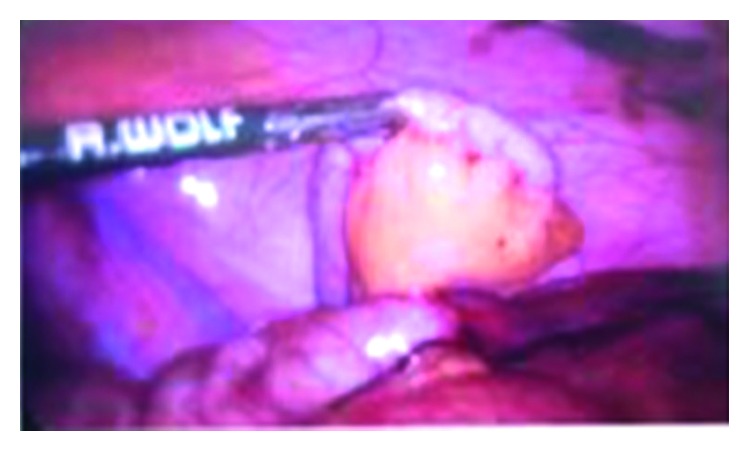
Appendix lifted with atraumatic laparoscopy forceps appears mildly congested.

**Figure 3:**
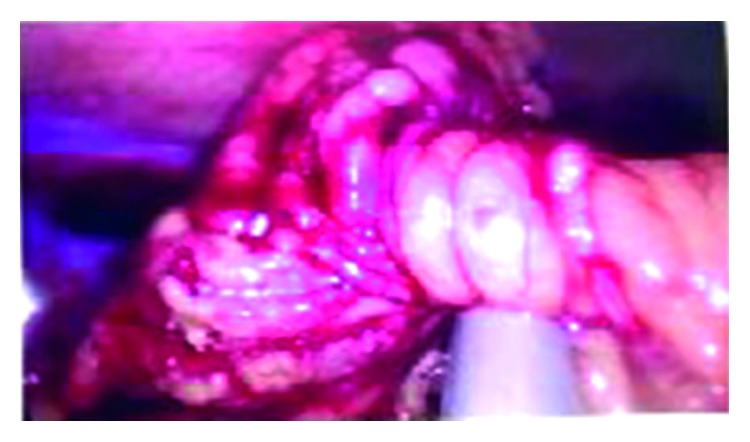
Torted omentum with a clockwise rotation.

He was observed, and after six hours, his abdominal pain persisted with increasing intensity. A decision was made to perform laparoscopy. At laparoscopy, the greater omentum was flimsily adhered to the right lower side of the abdominal wall, and there was a hemorrhagic area on the central part of the omentum with patches of pseudomembranes; the corresponding distal part (which was flimsily adherent to the abdominal wall) appeared necrotic, and small serosanguinous fluid was present in the pelvis. Figures 1–3 show some intraoperative pictures taken during the surgery.

An omentectomy was carried out using a harmonic. Due to the congestion of the appendix, a decision was made to remove the appendix at the same time. Postoperatively, he made a remarkable recovery and was discharged on the fourth day.

Histology report confirmed omental torsion (serosal fibrinous exudate containing acute and chronic inflammatory cells with marked fibrotic reaction and hemorrhagic congestion). Appendix showed early acute appendicitis (congested vessels with no obvious exudate).

## 3. Discussion

Torsion of the omentum is a rare cause of acute abdominal pain and may occur at any age. It is difficult to diagnose clinically in the preoperative setting. Accurate preoperative diagnosis was reported in the range of 0.6–4.8% [1]. Torsion of the omentum can be primary or secondary. In primary torsion, the distal end of the omentum is free, whereas in secondary torsion, the distal end is fixed to adhesions or some pathological condition. Primary torsion is said to occur when there is no pathological cause. It seems to occur more in the age group between 30 and 50 years (83 cases, 52.5%), and the male to female ratio is 2 : 1 [2]. However, Efthimiou et al. had reported a case of primary torsion in a 14-year-old boy among two cases [3].

Clinically, it presents as an acute abdomen with sudden onset pain that is localised in the right iliac fossa. Fever, nausea, and vomiting are infrequent presentations. Sixty-six percent (66%) of presentations resemble appendicitis, and twenty-two percent (22%) resemble cholecystitis [4].

Pathogenesis of primary omental torsion is not fully established but considered to be wide-ranged. Predisposing factors and precipitating factors have been reported. Predisposing factors include anatomic variations, obesity, and the arrangement of omental blood vessels [5, 6]. Precipitating factors include trauma, hyperperistalsis, and acute changes in the body position [7].

Preoperative diagnosis of primary omental torsion can be challenging. Basson and Jones analysed 223 cases of primary torsion and revealed that only one patient had been correctly diagnosed preoperatively [8]. Sometimes, ultrasonography and computed tomography can establish the diagnosis safely and allow conservative management. In the other ways, laparoscopy is a great help for the diagnosis and the treatment [9].

The current investigation tool and therapeutic management of choice is laparoscopy proceeding to laparotomy, identifying and removing the infarcted section of the omentum. Normal appendix, gallbladder, and fluid in the pelvic cavity make the diagnosis of omental torsion likely.

## 4. Conclusion

Omental torsion should be considered if preoperatively diagnosed acute appendicitis and Meckel's diverticulum were not found and if the gallbladder and ovaries reveal no disease. Secondly, the presence of serosanguinous fluid in the peritoneal cavity mandates inspection of the omentum to exclude torsion [1]. Awareness of omental torsion as a differential diagnosis for acute abdomen and a thorough inspection of the omentum in a negative laparoscopy are recommended for appropriate management [10].
